# Dataset of experimental and adaptive neuro-fuzzy inference system (ANFIS) model prediction of R600a/MWCNT nanolubricant in a vapour compression system

**DOI:** 10.1016/j.dib.2020.106316

**Published:** 2020-09-14

**Authors:** T.O. Babarinde, S.A. Akinlabi, D.M. Madyira, F.M. Ekundayo, P.A. Adedeji

**Affiliations:** a Department of Mechanical Engineering Science, University of Johannesburg, South Africa; b Department of Mechanical Engineering, Walter Sisulu University, Eastern Cape, South Africa; c Department of Industrial Engineering, Wayne State University, United States

**Keywords:** R600a, Experimental data, Power consumption, MWCNT nanolubricant, COP, Cooling capacity, ANFIS predicted data

## Abstract

This research paper assessed the performance of R600a with the base lubricant and Multi-walled Carbon Nanotube (MWCNT) nanolubricant at steady state. It describes the instruments required for measurement of the data parameter and its uncertainties, steps involved in preparing and replacing the MWCNT nanolubricant concentration with base lubricant in vapour compression refrigeration. The system's temperature data was collected at the components inlets and outlets. Pressure data was also registered at the compressor outlet and inlet. The data was captured at 27 °C ambient temperature at an interval of 30 min for 300 min. The experiment includes the experimental data collection, Adaptive Neuro-Fuzzy Inference System (ANFIS) training and testing dataset. The use of ANFIS model is explained in predicting the efficiency of MWCNT nanolubricant in a vapour compression refrigerator system. The ANFIS model also provides statistical output measures such as Root Mean Square Error (RMSE) and Mean Absolute Deviation (MAD), Mean Absolute Percentage Error (MAPE), and determination coefficient (*R*^2^). The data is useful and important for replacing MWCNT nanolubricant with base lubricant in a vapour compression refrigeration system for researchers in the specialisation of energy-efficient materials in refrigeration. The data present can be reused for vapour compression refrigeration systems simulation and modelling.

## Specifications Table

**Table:** 

Subject	Mechanical Engineering
Specific subject area	Nano materials for energy efficiency
Type of data	Text file, Figures, Tables
How data were acquired	The data collection source was from the mathematical equation and the experimental analysis. The measuring instruments used were Bordon pressure gauges, thermocouples (K-Types), mass flow metre, and electronic scales for weighing the mass of nanoparticles and refrigerant. A PC with MATLAB software was used in developing the performance prediction for the ANFIS model
Data format	Raw and Analysed
Parameters for data collection	The data collection parameters were the temperature of the refrigerant at the outlet of evaporator and condenser, the temperature and pressure discharge at the compressor and the refrigerant mass flow rate.
Description of data collection	The experiment's temperature was collected at the inlet and outlet of the refrigerator components (Condenser, Compressor, Evaporator, Expansion valve). The pressure at the outlet and inlet of the compressor were measure. The experimental data were captured at 27 °C ambient temperature at 30 min interval for 300 min by the data logger, the replication of the experiment was carried out five times to ensure precision. For the data training, 70% of the experimental data is used while the remaining 30% is used for the testing of the ANFIS model
Data source location	Department of Mechanical Engineering Science, Faculty of Engineering and the Built Environment,University of Johannesburg, Johannesburg, South Africa.
Data accessibility	With the article
Related research article	T. O. Babarinde, S. A. Akinlabi, and D. M. Madyira, “Energy performance evaluation of R600a/MWCNT-nanolubricant as a drop-in replacement for R134a in household refrigerator system,” Energy Reports, vol. 6, pp. 639–647, 2020. https://doi.org/10.1016/j.egyr.2019.11.132

## Value of the Data

• The data offers energy efficiency in the design and size of the nano eco-vapour compression refrigerator systems. The inadequate of actual raw experimental data greatly differentiate between the real experimental and expected predictive data value. The data value of the experiment provided in this experiment includes the anticipated data value on deviations and the estimated value of the ANFIS model prediction for the design and sizing of nano vapour compression refrigeration systems.
• The data describes steps involved for analysing the measured data and interpreting them, mathematical calculations involved in the analysis of nanolubricant performance which is useful to researchers and technicians in the area of cooling systems to substitute nanolubricant with the base or pure lubricant in vapour compression refrigeration systems.
• The ANFIS model offers the testing data to predict the efficiency of MWCNT nanolubricant in the vapour compression refrigeration system which is also useful in simulation and modelling by using other artificial intelligence approaches.
• The data improve and optimise concentrations of MWCNT nanolubricant in vapour compression refrigerator using R600a which can be useful in the application of other eco-friendly hydrocarbon refrigerants in vapour compression refrigerator systems.

## 1 Data Description

**Fig. 1:**
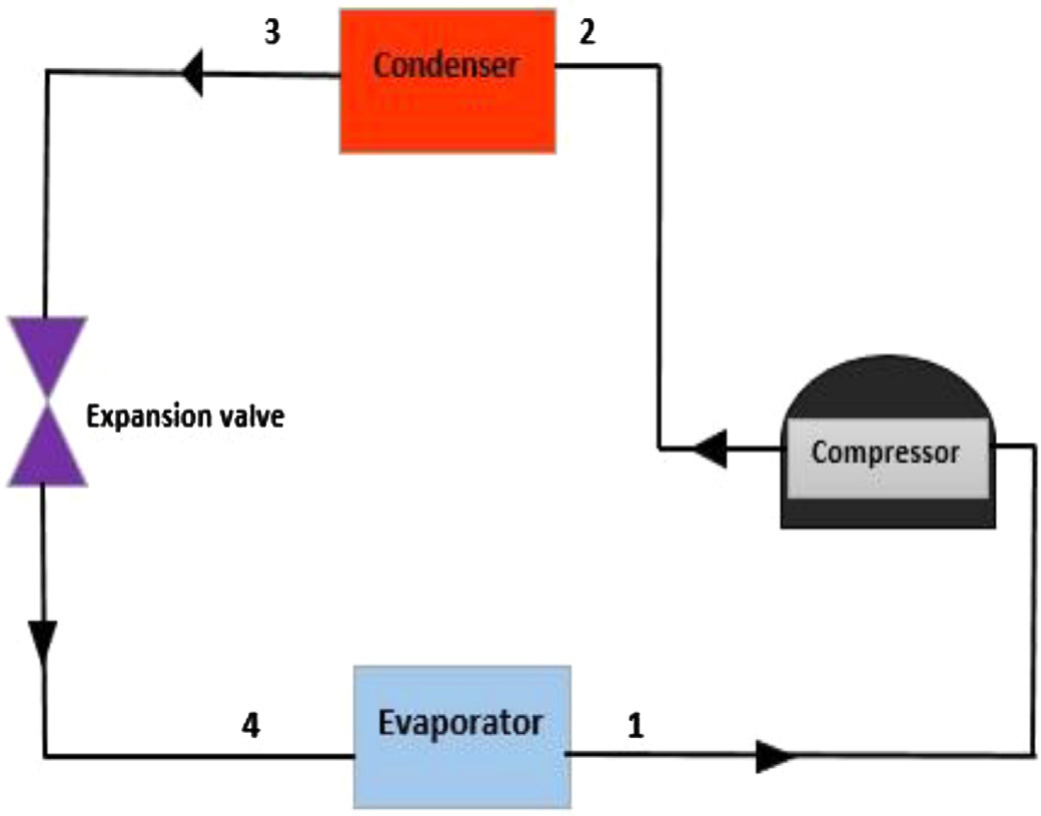
Diagrammatic representation of the test rig for the experiment.

**Fig. 2:**
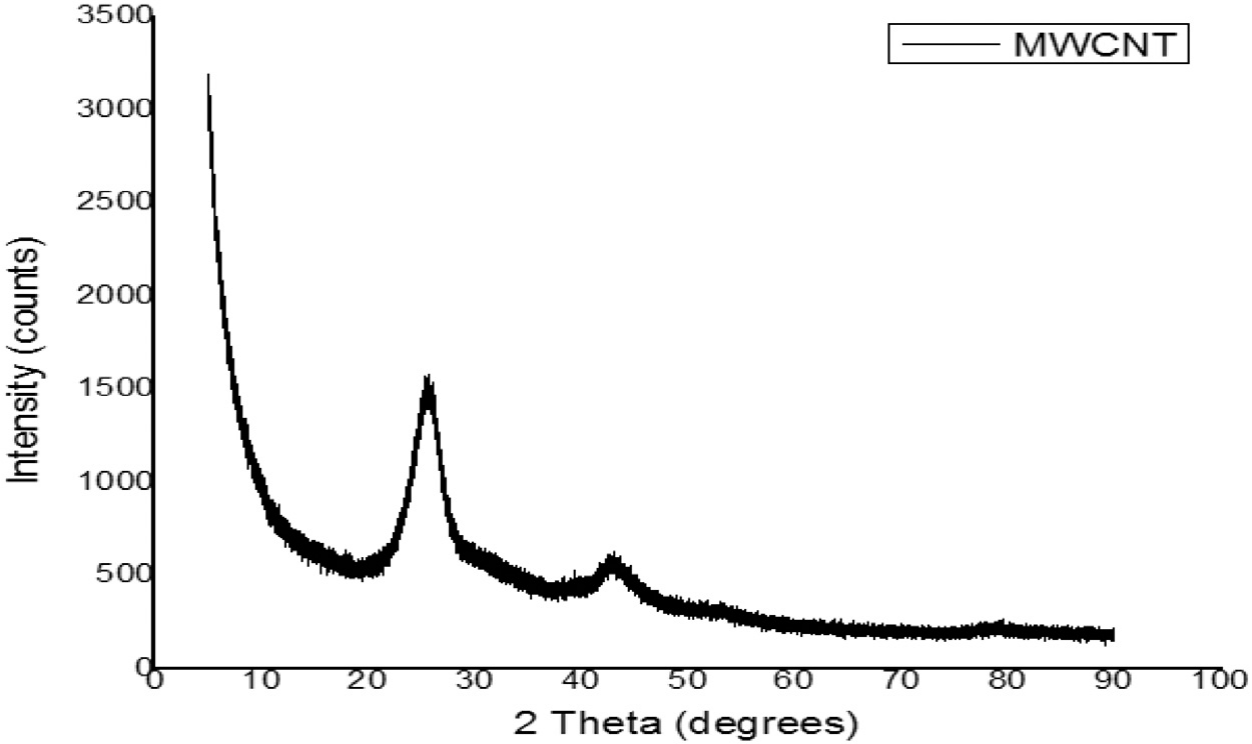
Structural integrity of MWCNT as revealed by XRD.

**Fig. 3:**
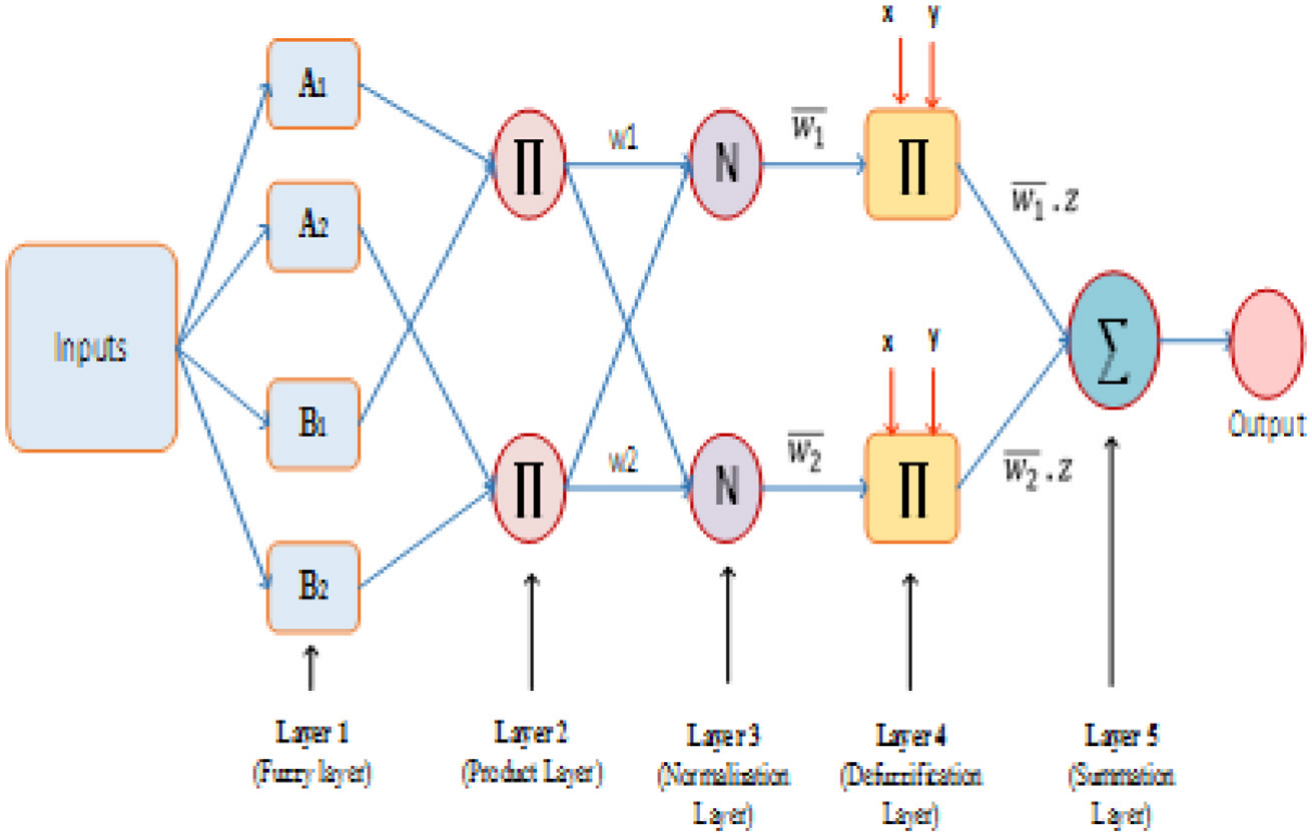
ANFIS architecture.

**Fig. 4:**
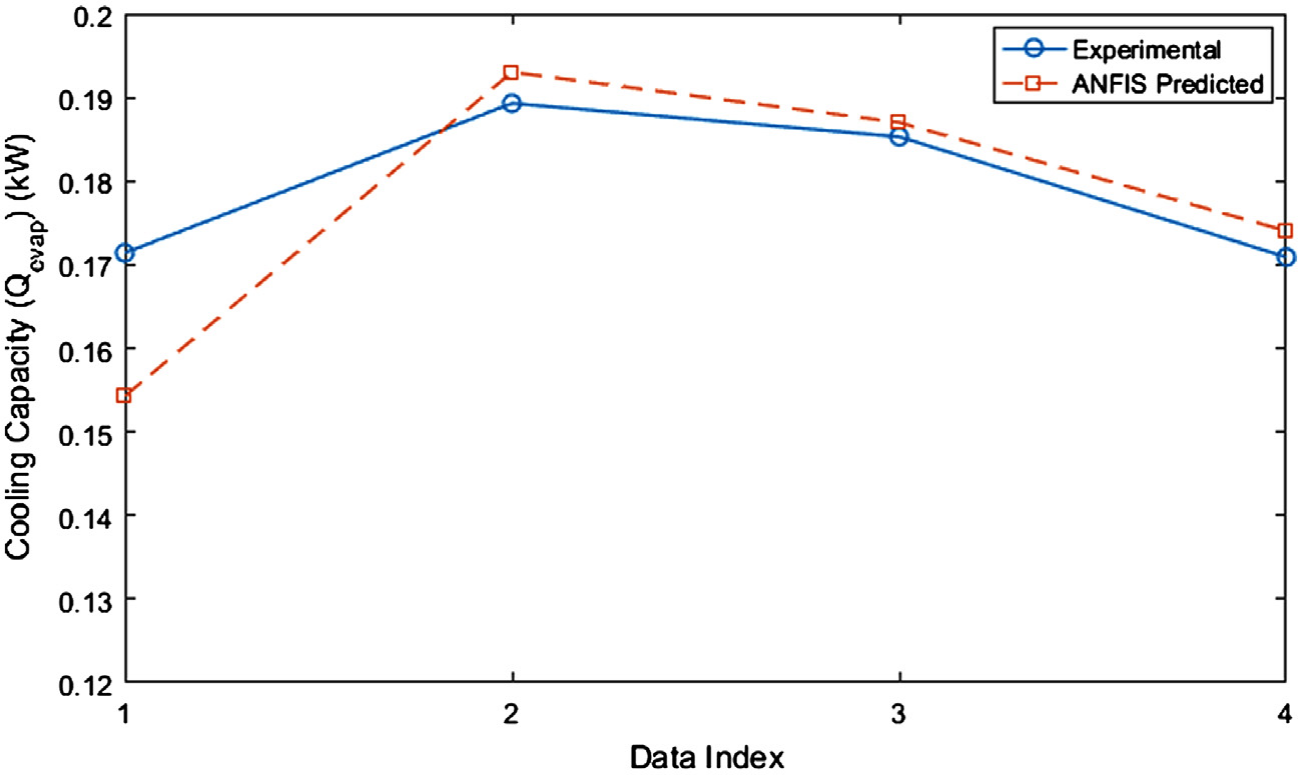
Comparison of the cooling capacity (Qevap) of experimental and ANFIS model predicted data.

The test rig diagram for experimental in Fig. 1 defines the inlet and outlet of each refrigerator component and the point of collection of each data. Fig. 2 revealed the structural integrity of MWCNT with the X-Ray diffraction. Fig. 3 illustrates the system's performance prediction ANFIS architecture with data values for input and output. Figs. 4 and 6 describe the performance comparison between the experimental and ANFIS predicted data of the MWCNT nanolubricant in the refrigerator system. Table 1 shows the dataset of the evaporator temperature and pull downtime of the refrigerator to achieve a steady-state performance. Table 2 present the data collection captured from different components test points of the system from the experiment which consist of R600a mass charge in gram (g), nanolubricant concentration (*N_c_*) in gram per litre (g/L), the temperature in degree Celsius (°C) at the condenser and evaporator outlet, pressure in kilopascal (kPa) at the compressor suction and discharge, and mass flow rate in gram per second (g/s) of R600a. Table 3 illustrates the R600a mass charge data collection and the enthalpy in kilojoule per kilogram (kJ/kg) of R600a with nanolubricant concentration of the evaporator, compressor, condenser, and mass flow rate. Table 4 shows the system output experimental data collection, the data set includes the system's R600a mass charge (g), nanolubricant concentration (g/L), compressor power consumption (*W_c_*) in (kW), evaporator chamber cooling capacity (Qevap) in (kW), and COP. Table 5 shows the system's experimental and ANFIS model predicted data set. Table 6 describes the statistical analysis of the predicted data set such as R-value, RMSE, MAD, and MAPE.

**Fig. 5:**
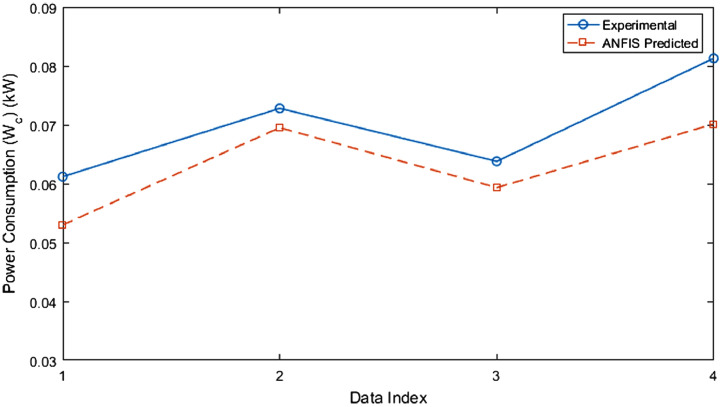
Comparison of the power consumption (Wc) of experimental and ANFIS model predicted data.

**Fig. 6:**
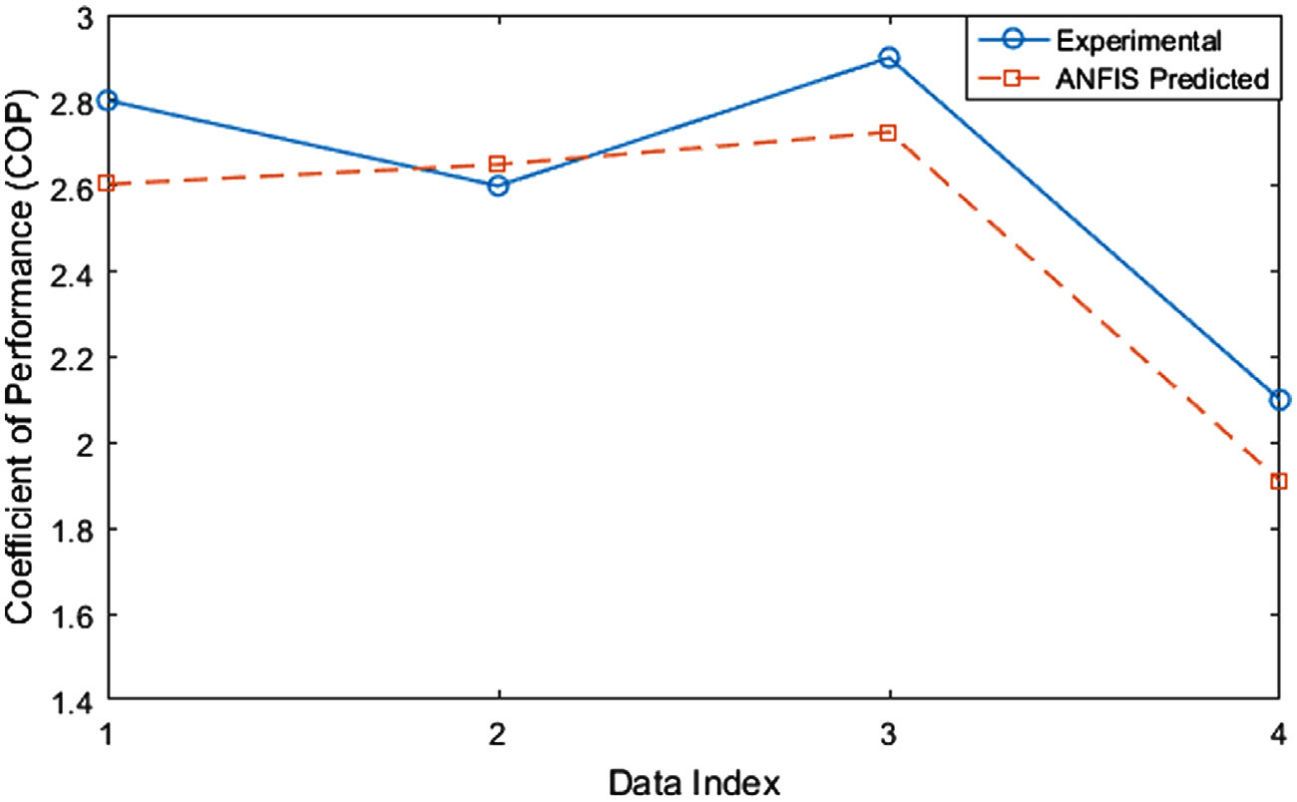
Comparison of the coefficient of performance (COP) of experimental and ANFIS model predicted data.

**Table 1:** Experimental dataset of the pulldown time and evaporator temperatures of the refrigerator at steady state.

Time (min)	0 (g/L)	50 g 0.2 (g/L)	0.4 (g/L)	0.6 (g/L)	0 (g/L)	60 g 0.2 (g/L)	0.4 (g/L)	0.6 (g/L)	0 (g/L)	70 g 0.2 (g/L)	0.4 (g/L)	0.6 (g/L)
0	27	27	27	27	27	28	27	27	27	27	27	27
30	9	7	0	−1	1	−2	−4	−3	5	5	5	1
60	2	0	−4	−5	−2	−6	−8	−5	2	1	−1	−3
90	−1	−2	−5	−6	−3	−6	−9	−7	1	0	−3	−5
120	−3	−4	−6	−6	−3	−7	−10	−8	1	0	−3	−5
150	−4	−4	−6	−7	−3	−7	−11	−9	0	−1	−3	−7
180	−4	−5	−7	−7	−3	−7	−11	−10	0	−1	−3	−7
210	−5	−5	−7	−8	−3	−7	−11	−10	0	−1	−3	−8
240	−5	−5	−7	−8	−3	−7	−11	−11	0	−1	−3	−8
270	−5	−5	−7	−8	−3	−7	−11	−11	0	−1	−3	−8
300	−5	−5	−7	−8	−3	−7	−11	−11	0	−1	−3	−8

**Table 2:** Experimental dataset of the collected pressure and temperature of the refrigerator at steady state.

Mass (g)	*N_c_* (g/L)	*T*_1_ (°C)	*P*_2_ (kPa)	*T*_3_ (°C)	*m_ref_* (kg/s)
50	0	−5	750	54	0.00052
50	0.2	−5	680	50	0.00053
50	0.4	−7	590	44	0.00056
50	0.6	−8	720	52	0.00057
60	0	−3	680	50	0.00052
60	0.2	−7	640	47	0.00059
60	0.4	−11	600	44	0.00062
60	0.6	−11	600	44	0.00060
70	0	0	750	54	0.00071
70	0.2	−1	750	54	0.00075
70	0.4	−3	740	53	0.00053
70	0.6	−8	660	48	0.00060

**Table 3:** Experimental dataset of the refrigerant's enthalpy at the steady-state in the refrigerator.

Mass charge (g)	*N_c_* (g/L)	*h*_1_ (kJ/kg)	*h*_2_ (kJ/kg)	*h_3_* (kJ/kg)	*ḿ_Ref_* (kg/s)
50	0	547.63	706.01	215.03	0.000522
50	0.2	547.63	687.65	225.57	0.000528
50	0.4	544.95	654.40	238.49	0.000559
50	0.6	543.61	669.41	216.51	0.000572
60	0	550.31	703.67	228.25	0.000521
60	0.2	544.95	681.57	230.71	0.000590
60	0.4	539.60	657.48	233.10	0.000618
60	0.6	539.60	645.28	233.10	0.000605
70	0	554.34	679.28	354.43	0.000705
70	0.2	552.99	664.10	352.99	0.000750
70	0.4	550.31	703.67	228.25	0.000531
70	0.6	543.61	670.29	226.90	0.000601

**Table 4:** Experimental dataset of the refrigerator's performance at steady state.

*N_c_* (g/L)	Mass (g)	Wc (kW)	Qevap (kW)	COP
0	50	0.0826	0.1736	2.1
0.2	50	0.0739	0.1700	2.3
0.4	50	0.0612	0.1714	2.8
0.6	50	0.0719	0.1870	2.6
0	60	0.0799	0.1679	2.1
0.2	60	0.0805	0.1853	2.3
0.4	60	0.0728	0.1893	2.6
0.6	60	0.0638	0.1853	2.9
0	70	0.0881	0.1410	1.6
0.2	70	0.0833	0.1500	1.8
0.4	70	0.0813	0.1709	2.1
0.6	70	0.0760	0.1902	2.5

**Table 5:** Dataset showing the experimental and ANFIS model predicted data.

Data Index	Experimental *W_c_* (kW)	ANFIS Predicted *W_c_* (kW)	Experimental Qevap (kW)	ANFIS Predicted Qevap (kW)	Experimental COP	ANFIS Predicted COP
1	0.0612	0.05301	0.1714	0.15426	2.8	2.604
2	0.0728	0.06951	0.1893	0.19301	2.6	2.650
3	0.0638	0.05934	0.1853	0.18701	2.9	2.726
4	0..0813	0.07014	0.1709	0.17403	2.1	1.911

**Table 6:** The ANFIS model dataset performance evaluation.

Variables	R2	RMSE	MAD	MAPE
Cooling capacity (Qevap)	0.692	0.02152	0.01635	0.0956
Power consumption (Wc)	0.302	0.00710	0.04910	0.0724
COP	0.883	0.28101	0.21190	0.0892

## 2 Experimental Design, Materials and Methods

A mineral oil and MWCNT nanoparticles were used to prepare the nanolubricant. The density of mineral oil is 0.914 at 15 °C and the viscosity is 32 cSt at 40 °C and 4.4 cSt at 100 °C. The MWCNT nanoparticles used in the lubricant as an additive have a diameter of 10 nm ± 1 nm ± 04,5 nm ± 0,5 nm ± 3–6 μm, the density of 0.068 g/cm^3^ as specified by the manufacturer (Sigma Aldrich). Every sample of MWCNT nanolubricant (0.2, 0.4, and 0.6 g/L) was prepared using a litre of mineral oil. The MWCNT nanoparticles and lubricant were stirred together with the help of magnetic stirrer for 45 min. After that, the ultrasonic homogeniser was then used to homogenised MWCNT nanolubricant together under a temperature range of 15–20  °C for 180 min [1,2].

The used R600a refrigerant has GWP of 3 and ODP of zero. The refrigerator that served as the test rig for the experiment was a 70-l capacity household vapour compression system with 100 W power ratings hermetic compressor, expansion valve, 9.8 m length air-cooled condenser and 1.5 m length capillary tube.

Each concentration of nanolubricants was measured for 50, 60, and 70 g. The refrigerant (600a) mass charge with charging scale was inserted into the system compressor. Evacuation and flushing of the system were also performed to ensure greater data accuracy for each experiment. The temperature measurements were taken with thermocouples at each inlet and outlet of components for the refrigerator. The two pressure gauges were attached to a compressor to calculate the compressor's suction and discharge pressure.

The experimental test was conducted and repeated at an ambient temperature of 27 °C for 300 min at an interval of 30 min for 5 times. Thermocouples and pressure gauges instrument uncertainties were respectively +3 °C and 1 %. Fig. 1 depicts an experimental set-up schematic-diagram. Using Refprop version 9.0 [3], the experimental data output readings were used to determine system performance.

Consideration was given to performances such as power consumption (Wc), cooling capacity (Qevap), and COP.

The efficiency of R600a in MWCNT nanolubricant, such as power consumption (Wc), cooling capacity (Qevap), and COP, was analysed according to Madyral et al. [4] using Eqs. (1)–(3)

$\overset{\prime }{m}$ The Q evap was computed with Eq. (1)

(1) $$Q_{evap}=\overset{\prime }{\underset{Ref}{m}}{\left(h_{1}-h_{4}\right)}_{evap}$$

Where *h*_1_, *h*_4_ and ḿref represent enthalpy of refrigerator evaporator at outlet, inlet and mass flowrate, respectively. The condenser's enthalpy outlet (*h*_3_) at steady state is equal to enthalpy of the evaporator's outlet (*h*_4_). Hence, *h*_3_ = *h*_4_

The calculation of *W_c_* result data follows Eq. (2)

(2) $$W_{c}=\overset{\prime }{\underset{Ref}{m}}{\left(h_{2}-h_{1}\right)}_{c}$$

Where the compressor outlet enthalpy is *h*_2_

The *COP* data is calculated utilizing Eq. (3)

(3) $$COP=\frac{Q_{ev}}{W_{c}}$$

ANFIS modelling technique is a five-layer feed forward network that combines ANN and Fuzzy logic technology to build a hybrid model capable of practical fit and efficient performance in a system where imprecision and uncertainty can exist. Within ANFIS, all nodes are adaptive for both the first and fourth layers while other layers have non-adaptive nodes. The model follows the Fuzzy Inference System Takagi Sugeno (TSFIS). The input data for these model are evaporator temperature (*T*_2_), condensing temperature (*T*_3_), refrigerant mass charge and nanolubricant concentration (*N_c_*). As stated earlier, 70% of the data was used for training while 30% of the data was used in the model testing. The ANFIS MATLAB model program can be found online in the supplimentary file. The rules of the ANFIS model are established by an if-then principle that connects the precedent with the consequence as follows:

Condition 1: If *X*_1_ is A_1_ AND *X*_2_ is *B*_1_ then *f*_1_ = *r*_1_*X*_1_ + *s*_1_*X*_2_ + *t*_1_.
Condition 2: If *X*_1_ is A_2_ AND *X*_2_ is *B*_2_ then *f*_2_ = *r*_2_*X*_1_ + *s*_2_*X*_2_ + *t*_2_.

Where *X*_1_ and *X*_2_ are model inputs, the parameters of *A*_1_ *A*_2_,_1_ and *B*_2_ are fuzzy and r, s, t.

The model for the ANFIS structure is shown in Fig. 4. The parameterisation for each layer is as follows:

Layer 1: This layer consists of adaptive nodes with fuzzy membership function which calculates the output function by

(4) $$O_{j}^{1}=\mu_{Aj}\left(I_{1}\right),j=1,2$$

(5) $$O_{j}^{1}=\mu Bj\left(I_{2}\right),j=1,2$$

Layer 2: The nodes in this layer are non-adaptive and, according to Eq. (9), the firing power of each law is measured from a multiplicative operator.

(6) $$O_{j}^{1}=\mu_{A_{j}}\left(I_{1}\right).\;\mu_{B_{j}}\left(I_{2}\right),\;j=1,2$$

Layer 3: Nodes in this layer are also set with the normalisation of the firing strength ${\bar{\bar{w}}}_{i}$ in the *j*th node executed using the ratio of its firing strength and the combination of all firing strengths according to all rules.

(7) $$O_{j}^{3}{\bar{w}}_{j}=\frac{w_{j}}{w_{1}+\;w_{2}},\;j=1,2$$

Layer 4: Nodes in this layer are adaptive, and defuzzification is performed. The effect of the *j*th rule on the output of the layer is expressed here according to Eq. (12) and node parameters are defined by *r_j_,s,t_j_*.

(8) $$O_{j}^{4}={\bar{w}}_{l}\left(r_{j}I_{1}+s_{j}I_{2}+t_{j}\right)={\bar{w}}_{l}z_{j}$$

Layer 5: This layer consists of non-adoptive nodes that combine all incoming signals from the previous layer by a summing function

(9) $$O_{j}^{5}=\;\sum_{j}\bar{w_{i}}z_{j}\;=\;\frac{\sum_{j}w_{j}z_{j}\;}{\sum_{j}w_{j}}$$

The network has been tested according to Adedeji et al. [5]. ANFIS predicted values and statistical performance metrics such as determination *R*^2^, RMSE, MAD and MAPE were compared to the experimentally determined model outputs. Those measurements were determined as follows:

(10) $$R^{2}=1-\frac{\sum_{k=1}^{N}\left[y_{k}-\hat{y_{k}}\right]}{\sum_{k=1}^{N}\left[y_{k}-\bar{y_{k}}\right]}$$

Root Mean Square Error (RMSE)

(11) $$RMSE=\;\sqrt{\frac{\sum_{k=1}^{N}{\left[y_{k}-\;\hat{y_{k}}\right]}^{2}}{N}}\;$$

Mean Absolute Deviation (MAD)

(12) $$MAD=\;\frac{1}{N}\;\sum_{k=1}^{N}\left|y_{k}-\;\bar{y}\right|$$

Mean Absolute Percentage Error (MAPE)

(13) $$MAPE=\;\frac{1}{N}\;\sum_{k=1}^{N}\left|\frac{y_{k}-\;\hat{y_{k}}}{y_{k}}\right|\times \;100\%$$

Where the observed value is *y_k_*, ${\hat{y}}_{k}$ is predicted value, and $\bar{y}$ is the observed mean

## Declaration of Competing Interest

None.

## Appendix Supplementary Materials

Image, application 1

Image, application 2

Image, image 3

Image, image 4

Image, image 5
