# Supplementary figures and images for: Dataset of experimental and adaptive neuro-fuzzy inference system (ANFIS) model prediction of R600a/MWCNT nanolubricant in a vapour compression system
Source: Data Brief. 2020 Sep 14;32:106316. doi: 10.1016/j.dib.2020.106316 (PMC7516065; doi:10.1016/j.dib.2020.106316)

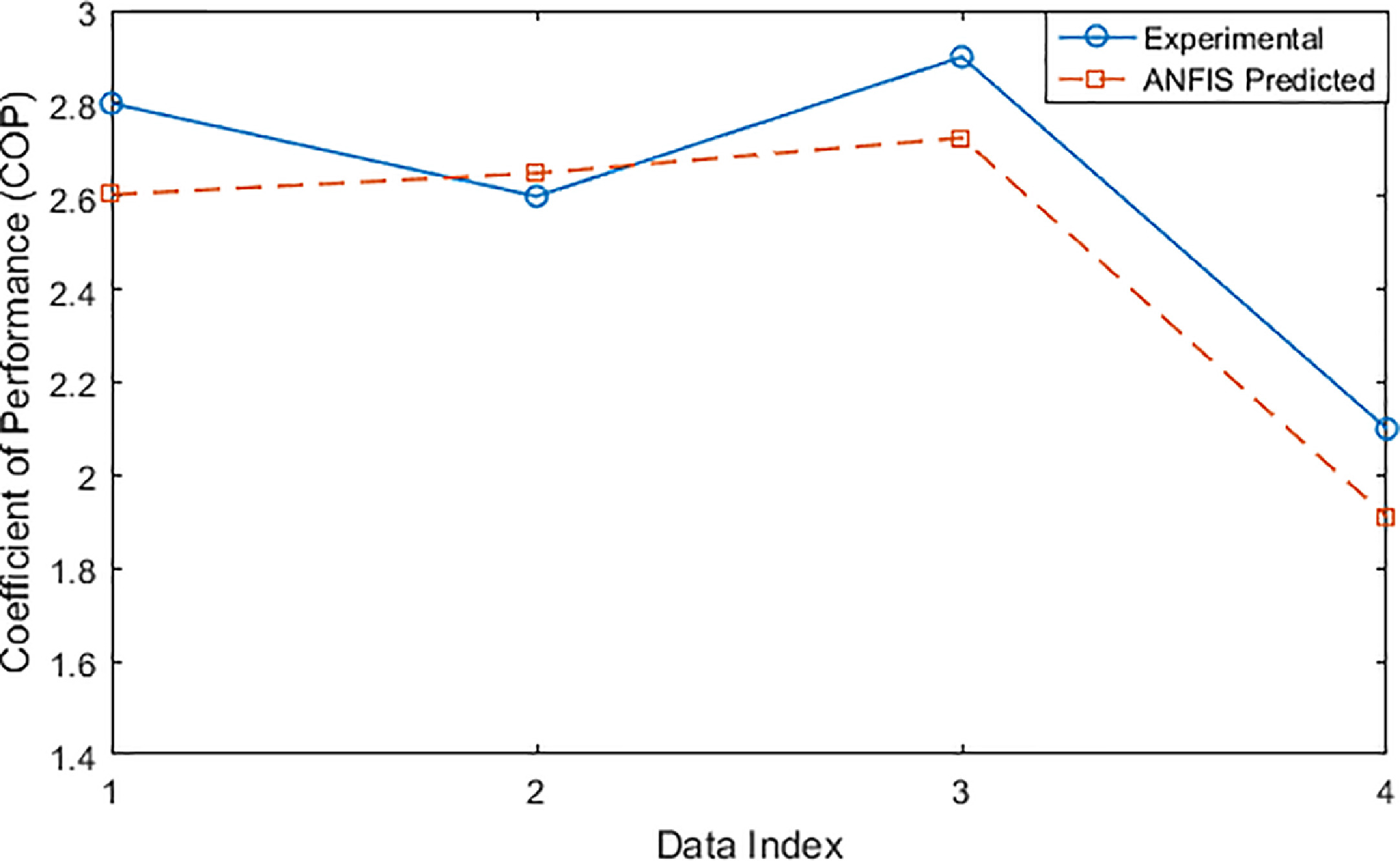

Supplement: Supplementary file 3 [file mmc3.jpg]

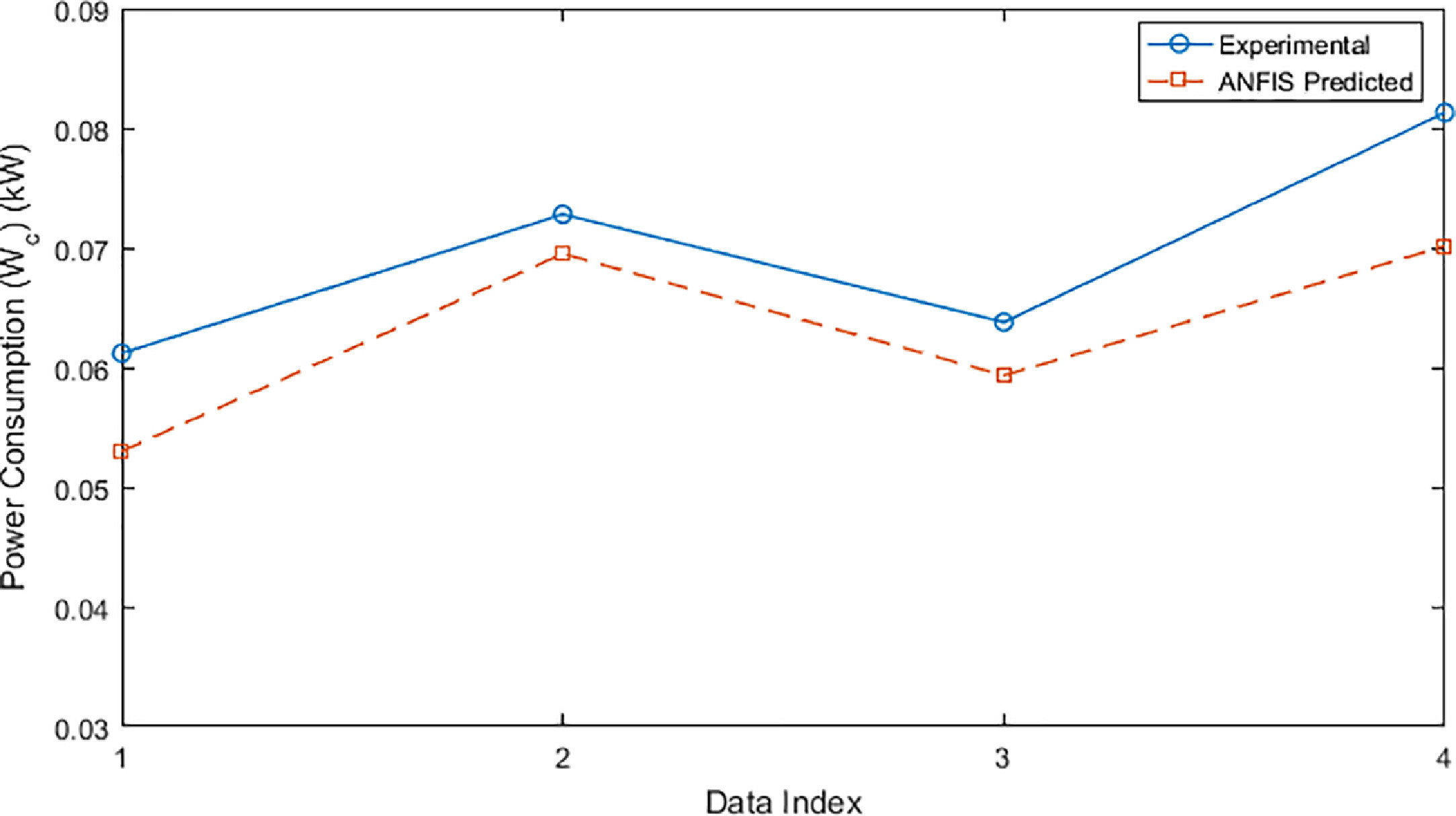

Supplement: Supplementary file 4 [file mmc4.jpg]

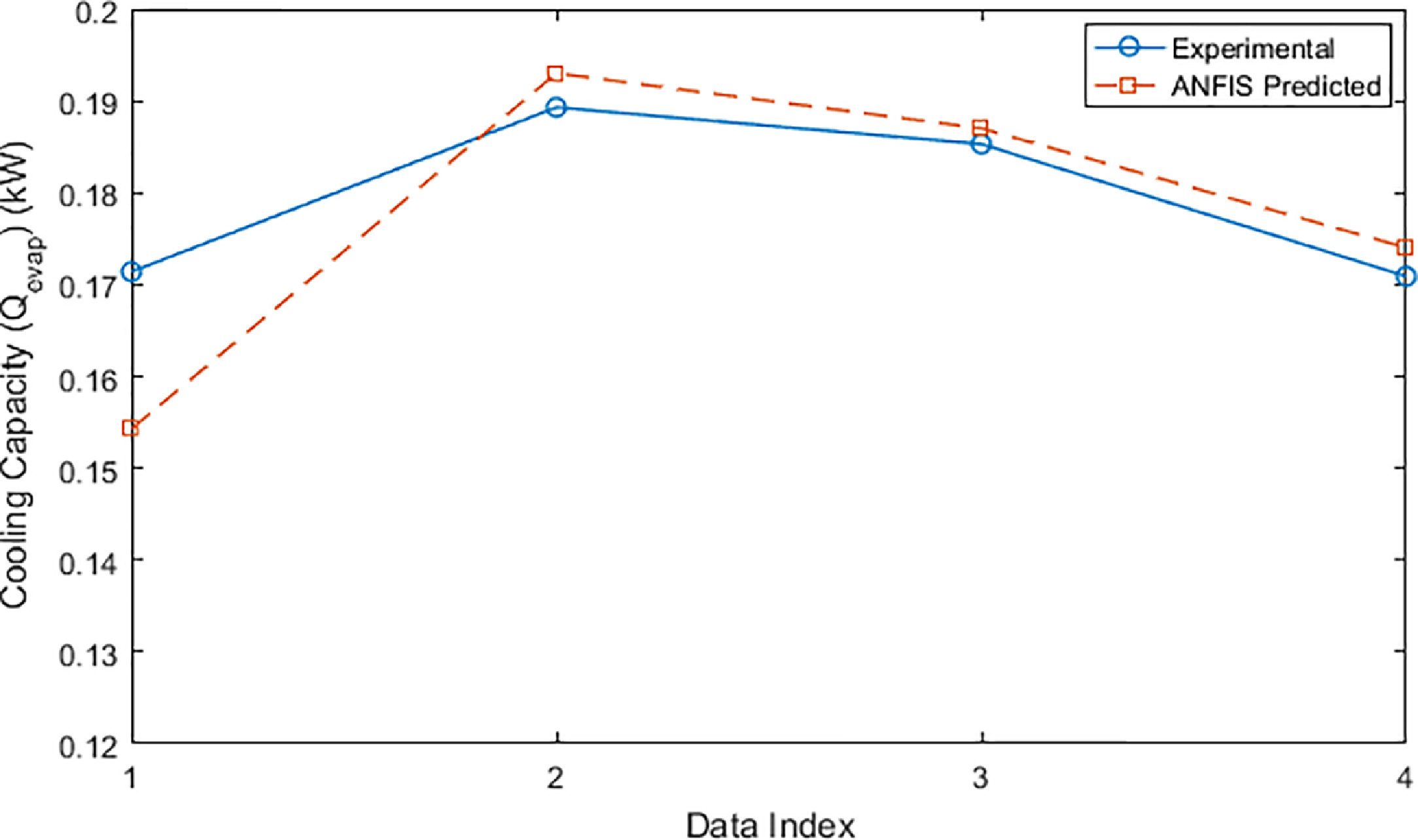

Supplement: Supplementary file 5 [file mmc5.jpg]
